# “It’s a walk of shame”: Experiences of unintended pregnancy and abortion among sexual- and gender-minoritized females in urban India

**DOI:** 10.1177/23992026211027698

**Published:** 2021-07-31

**Authors:** Jessamyn Bowling, Megan Simmons, Donna Blekfeld-Sztraky, Elizabeth Bartelt, Brian Dodge, Vikram Sundarraman, Brindaa Lakshmi, Debby Herbenick

**Affiliations:** 1 Department of Public Health Sciences, University of North Carolina at Charlotte, Charlotte, NC, USA; 2 Indiana University, Bloomington, IN, USA; 3 University of Buffalo, Buffalo, NY, USA; 4 Nirangal, Chennai, India

**Keywords:** LGBTQ, India, abortion, contraception, lesbian, bisexual, sexual & gender minority (SGM)

## Abstract

**Background:**

Unintended pregnancy and safe abortion access in India remain critical public health concerns. The health of sexual- and gender-minoritized females (SGMF; those assigned female at birth and identify as other than heterosexual and/or as other than cisgender women) in India is understudied.

**Aim:**

We examined experiences of unintended pregnancy and abortion among SGMF individuals in urban India.

**Methods:**

We used focus group discussions (*n* = 8 individuals in two groups) and interviews (*n* = 20) with SGMF individuals. Data were collected in December 2017. Transcripts were analyzed using a priori thematic analysis and then open thematic analysis in Dedoose online software.

**Results:**

Nine participants experienced or suspected they had unintended pregnancies. Pregnancy circumstances were mostly due to sex without using a barrier method. Participants discussed using traditional methods to induce abortion or changing their approach to contraception. Social support was often lacking, though partners were supportive of abortion choices. Participants reported stigma and surveillance from family, friends, providers, and community members.

**Conclusion:**

These findings highlight the effects of stigma in relation to abortion and unintended pregnancy on health and relationships.

## Introduction

Universally, unintended pregnancy and safe abortion access remain critical public health concerns. Unintended pregnancy often carries stigma,^1^ associated with challenging decisions regarding parenting, adoption, or abortion.^2,3^ On a global scale, abortion rates for upper middle-income countries have consistently declined over the past 20 years, and lower middle-income countries have continued to witness a steady increase. Many of these abortions can be attributed to the lack of primary reproductive health services linked with expanding populations in rural areas.^4,5^ In 2015, researchers found that approximately half of all pregnancies in India were labeled as “unintended” with nearly one-third of all pregnancies ending in abortions, equating to about 15.6 million per year.^5^

In 1971, abortion was legalized in India through the Medical Termination of Pregnancy Act (MTP Act), which authorizes abortions up to 20 weeks of pregnancy with specific limitations (including physical injury, mental health, in cases of economic or social necessity, in cases of rape for married women, or likely physical or mental disabilities for the child).^6^ Another study from India found that only 31% of reported reasons for abortion fell under the guidelines laid out by the MTP, while the vast majority cited that the pregnancy was unwanted.^7^

Abortions among unmarried women in particular appear to be relatively common. A study out of northeast India found that nearly three-quarters of women opting for a first-time abortion were unmarried and seeking abortion due to socioeconomic reasons.^8^ Unmarried women in India seek abortions for a variety of reasons, including the negative perception and social stigma associated with pregnancy outside of marriage, experiences of domestic violence, costs associated with childbearing, work-related pressures, and desires to continue education.^9–11^ Abortions outside of marriage were often characterized by the need for secrecy and perceived stigma, particularly among young people, as minors are required to get guardian consent for the procedure under the MTP.^12,13^ For unmarried women over the age of 18, lack of partner or family support may exacerbate access barriers if providers insist on obtaining consent from relatives or the other person involved in the pregnancy.^9,11,13–16^

Given the pervasive stigma around unintended pregnancy outside of marriage, younger women are also more likely to identify and disclose their pregnancies later in gestation, given amid fear of social repercussions.^9,11,13,16^ Unmarried women are more likely to access abortion later in pregnancy, to utilize informal providers, and abortion self-management (with limited options for self-management later in pregnancy) due to their concerns regarding confidentiality.^9,17^ Barriers such as lack of mobility and lack of financial resources may also influence the decision to seek abortion outside medical settings.^13^ Characteristics such as being older and having more formal education are associated with obtaining abortion earlier in pregnancy among unmarried women.^11^

Beyond policy barriers, clinics and providers may act as gatekeepers for abortion in India. Although spousal or familial consent is not legally required for adults seeking abortion in India, studies have documented medical providers create and enforce their own rules regarding external consent for abortion.^7,14^ Other studies have documented stories from married women wherein providers were reluctant to perform later abortions due to concerns about sex selective motives, suggesting further potential barriers exacerbated by the medical community.^7^

Despite the passage of the MTP Act, people in India may use alternative methods than medical abortions as they navigate existing barriers and stigma. Those who lack access to abortion through trained and licensed doctors and safe medical facilities may turn to pharmacists to procure medication kits (typically consisting of mifepristone and misoprostol) to self-manage their abortion at home.^26^ Due to these drugs availability, many people depend on these medications to terminate their pregnancy without first consulting a physician. Life-threatening complications are not uncommon in people administering these drugs by themselves.^27^ Banerjee et al.^18^ focused on 381 women in India with post-abortion complications and found that 53% of these women first attempted a self-induced abortion. Of those women, 95% had related complications.

Alternately, people may seek unqualified vendors of medication or use traditional medicine to induce and self-manage abortion.^5^ Other reasons for self-induction included avoiding the logistical issues and inconvenience associated with traveling to a hospital for an abortion procedure, as well as the ability to discreetly terminate a pregnancy without having to explain absences from the home to relatives.^26^

Despite a body of research examining unintended pregnancy and abortion experiences in Indian women, previous work has yet to examine experiences of unintended pregnancy and abortion among sexual- and gender-minoritized females (SGMF; i.e. those assigned female at birth whose sexual identity is other than heterosexual, such as lesbian, bisexual, and/or whose gender identity is other than cisgender woman, such as genderqueer or nonbinary). Evidence from industrialized Western countries indicates higher rates of unintended pregnancies among SGMF compared with heterosexual female individuals.^19^–^21^ Homophobia, transphobia, cisnormativity, and heteronormativity have been cited as driving negative health outcomes among SGMF people for many common reproductive health issues (e.g. pap screenings, cancer rates).^22,23^ Studies examining abortion experiences among transgender and gender expansive people in the United States indicate that identity-related denial or care and mistreatment by medical practitioners leads some people to consider or attempt self-managed abortion.^24^ Self-management through the use of medication, or in some cases use of herbs, was also perceived to be less invasive and to offer more privacy than clinic-based abortion.^25^ This may be particularly salient in settings that do not have competency in transgender or gender-inclusive health care.^28^

Research with SGMF in India has documented how stigma related to sexual and/or gender identity affects some aspects of reproductive health and sexual autonomy, including family formation, intimate and family relationship support, and access to contraception.^29–31^ However, studies have yet to examine this in relation to abortion. Although it is likely that some issues surrounding unintended pregnancy and abortion among SGMF people in India apply to females in general (e.g. reasons for abortion, abortion stigma), SGMF individuals in India may be subject to additional barriers to access, including identity-based discrimination and isolation. This study seeks to examine experiences of unintended pregnancy and abortion among SGMF in urban India.

## Materials and methods

This is a multi-method qualitative study using focus group discussions and interviews. We partnered with two community organizations, Nirangal in Chennai and Kolkata Rista in Kolkata. Community partners collaborated on protocol development, participant recruitment, data collection, analysis, and dissemination. Recruitment messaging advertised a study about contraception and sexuality. We conducted two focus group discussions with SGMF in Bangalore (*n* = 4) and Chennai (*n* = 4) in December 2017. We conducted semi-structured in-person interviews with SGMF in Bangalore, Chennai, and Kolkata (*n* = 20). Eligibility criteria included being at least 18 years of age and identifying as something other than “heterosexual” and/or “cisgender.” Because of our focus on pregnancy and abortion for the present analyses, this article focuses on participants who were assigned female at birth, though the full study included those who were assigned male at birth as well. Participants were recruited through community partners’ social media and word of mouth in a convenience sample, and were compensated with 500INR (about US$8). No participants dropped out, though due to passive recruitment we do not know how many participants saw the recruitment but did not participate.

Before the study, participants did not know the US-based team members but knew at least one community partner. Participants were told that the researchers were from a US university, had conducted previous research with SGM individuals, and were interested in how SGM individuals in India were thinking about sexuality. All participants gave verbal consent to proceed to the interview or focus group, as the research team’s previous experience with SGM research in India included fears of stigma related to identity disclosure with written names. The Institutional Review Board at Indiana University, Bloomington approved all research protocols (#1710732738).

Both the interviews and focus groups were conducted in participants’ preferred language (Bangali, English, Hindi, and/or Tamil) in community venues such as SGM community centers or a rented conference room with only participants and researchers present. Focus group discussions and interviews were co-facilitated/conducted by US-based researchers with community partners (J.B., M.S., V.S., B.L.), lasting between 30 and 90 min. Team members based in the United States were all white, cisgender, and born in the United States. Team members based in India were all gender and/or sexual minority individuals from India. Nearly all team members had conducted research with SGM individuals in India previously. Participants selected their own pseudonyms which will be used in this article. (We use “they/them/theirs” when referencing individual participants because of unknown or inconsistent pronoun collection.) Both methods were audio-recorded and translated and transcribed; field notes were collected which informed probes for subsequent interviews. We used Dedoose online qualitative software^32^ to analyze the data, with each transcript coded by two coders from the full team of four coders. A priori coding based on interview and focus group discussion guides was used first, followed by open coding of additional themes from the collected data.^33^ Interrater reliability was confirmed through the “test” function of Dedoose. Codes with a Cohen’s Kappa less than 0.80 were discussed and refined by the coding team. The team then conducted inductive thematic analyses by grouping common concepts to form themes.^33^ See Table 1 for the coding tree of relevant themes. Coders used the memo function in Dedoose, or electronic notes attached to excerpts; these informed discussions of bias, influenced by social locations and personal experiences.^34^

**Table 1.** Coding tree related to unintended pregnancy, abortion, and pregnancy scares.

Unintended pregnancy experiences and scares	Circumstances of realizing pregnancy	Personal reactions
		Lack of knowledge of options and access to services
	Decision making	Social support
		Access to services
		Anticipated judgment

## Results

Among the 28 participants, nine participants reported experiences with unintended pregnancies or unconfirmed/suspected pregnancies (see Table 2 for included participant demographic information). The recency of unintended pregnancy experiences ranged from a few months to years prior to participation in the study. Participants reflected on the circumstances in which they became pregnant, emotional and behavioral reactions to the pregnancy or suspected pregnancy, pregnancy decisions and abortion care seeking, and barriers to care, which occurred across personal, interpersonal, organizational, and cultural levels. Participants described negative emotional reactions upon realizing they were pregnant, were often unaware of their options (e.g. methods of abortion), faced isolation and judgment within their social networks and in health care environments.

**Table 2.** Sexual and gender minority female participant demographics (*N* = 28, 9 with experiences of unintended pregnancies).

Focus Group Discussions (*n* *=* 8 participants; 1 with unintended pregnancy and/or abortion experience(s))				
Pseudonym	Age	Gender identity	Sexual identity	Relationship status
Chennai Women FGD				
Chennai W2	24	I am largely a woman	Non-heterosexual	Single, not dating
Interviews (*n* *=* 20; 8 with unintended pregnancy and/or abortion experience(s))				
Chennai Interviews				
Aquafina	27	Non-conforming	Pansexual	Committed relationship
Karla	23	Female	Bisexual	Committed relationship
Annette	28	Cisgender female	Pansexual	Committed relationship
Shivvie	–	Genderfluid	–	Committed relationship
Bangalore Interviews				
Pooja	22	Genderfluid	Homosexual	Committed relationship
Asma	23	Female	Bisexual	Committed relationship
Kolkata Interviews				
Pompi	29	Genderfluid	Pansexual	Committed relationship, polyamorous
Jia	42	Nonbinary	Pansexual	Married

FGD: focus group discussions.

### Pregnancy circumstances and personal reactions

Participants discussed their contraception (or lack thereof) with partners at the time that they realized they were pregnant. Finding out about pregnancy caused negative emotional reactions as well as actions to prevent future unintended pregnancies. Lack of condom use was attributed to a variety of factors, including lack of access, getting caught up in the moment, sexual coercion, and preference for other methods of contraception. (For more information on participants’ reasons for and against contraceptive use, see Simmons et al., 2020.) Reflecting on a budding relationship, Asma related the fear they and their partner experienced after they got caught up in the moment during sex and failed to use a condom (Table 3, 1.1a). Having been accustomed to using withdrawal as a primary method of contraception, Jia noted their surprise at morning nausea (Table 3, 1.1b). After being encouraged to take two home pregnancy tests by their friends, Jia called their boyfriend with the news of the pregnancy, who laughed (Table 3, 1.1c).

**Table 3.** Example quotations with participant pseudonym, location, age, gender identity, sexual identity.

1.1 Pregnancy Circumstances and Personal Reactions	(a) “Last month only [I was afraid I was pregnant]. In between, we were so much into each other so we did not have condoms at home, so we just ended doing [sex], so we were a bit scared . . . my period shifted a little bit late.”—Asma (Bangalore, 23 years, female, bisexual)
	(b) “I was sure it [semen] didn’t go in . . . which is why I didn’t think I was pregnant at all.”*—*Jia (Kolkata, 42 years, non-binary, pansexual)
	(c) “I tried [a second test], that also turned out positive and I called him up and I’m telling him this and he’s laughing . . . I started crying ’cause I didn’t know what to do and I was too young and I was like ‘No I cannot have a baby’”.*—*Jia (Kolkata, 42 years, nonbinary, pansexual)
	(d) “The second time that it had happened it was more like a moment saying, ‘I can’t believe this. Again. Again?’ The second time was also like, ‘I can’t even talk about this anymore, because how hopeless am I?’”—Aquafina (Chennai, 27 years, non-conforming, pansexual)
	(e) “Pompi: When I was 16 years old [I was worried I was pregnant]. So at that point in time, the boyfriend I had, he came inside me without a warning and then I had to take an [emergency contraception] pill 18 hours later, and that messed up my balance like that. So that was the only time that I have taken emergency contraception. That experience has been so scary that I never want to go back. Interviewer: Yeah. Did that experience change the way that you were having sex moving forward? Pompi: Yeah. Because until then, using a condom was not a must for me.”—Pompi (Kolkata, 29 years, genderfluid, pansexual)
	(f) “I’ve been on the pill since I was like 16, because I’m not taking any chances. And I’ve been there, in spite of being on the pill, I’ve had to have an abortion and when I was doing my Master’s, so I was like 23. This was with somebody who was my boyfriend at the time. And it was a messed up situation and not want to go in there ever, [have an abortion]. That’s there, but then like I said, accidents can still happen and . . . especially with male-bodied people, I’m very, very, very insistent on condoms”—Jia (Kolkata, 42 years, nonbinary, pansexual)
	(g) “[If I got pregnant with my fiancé] it’s [my relationship is] gonna be still there. The thing that I’ll know when I might get pregnant and I can just go ahead and abort and just do the same cycle that I did before, and because he’s also my fiancé, there’s a [reassurance] attached to it of me knowing my cycle and me knowing when I might get pregnant, when I might not, so I think there’s more than one person looking out to know . . . So there’s a lot more work involved to not get me pregnant.”—Karla (Chennai, 23 years, female, bisexual)
1.2 Pregnancy Options	(a) “I took the home pregnancy test and figured I was pregnant. I didn’t know where to go. Didn’t know what to do.”—Aquafina (Chennai, 27 years, non-conforming, pansexual)
	(b) “When I checked it, it showed positive, and I believed that I’m going to have the child. Like, I made some crazy decisions. I was thinking that I’m going to actually have the child. I didn’t know any other option back then [other than to have a child]. I was very young at that time, and I was so naïve thinking that he’s going to come back and tell my family, and we are going to get married. But, none of that happened. But then, after some two to three weeks, I started bleeding, and it went on to a natural abortion.”*—*Shivvie (Chennai, genderfluid)
	(c) “I thought I had an almost pregnancy, so I kind of saw the signs quite early and just self-aborted at home with a lot of just increasing body heat . . . If you want to get your period, it’s all in the Hindu culture. I’m from a Hindu family, so have papaya, have some cinnamon water. It all really helps to get the body heat up and there’s a lot of oils . . . just to increase the body heat. These are all quite natural practices in India, in my culture, at least. The more you increase the body heat, the more you’re sure to bleed out . . . around and if they want to be done with their bleeding cycle and all of that.”*—*Karla (Chennai, 23 years, female, bisexual)
2. Social Support (Or Lack Thereof)	(a) “I skipped my periods, and he [my partner] was not here. I had to buy the kit to check if I’m pregnant. I had absolutely no idea. I was so young back then, so I googled a lot. I was so scared. I couldn’t tell anybody. There was absolutely nobody I could confide at that time.”*—*Shivvie (Chennai, genderfluid)
	(b) “I also didn’t know who to reach out to. Because there was also lots of older people in my life, all very supportive, all saying, ‘Don’t worry about it. We’ll get this sorted’ . . . People I’ve worked with. Bosses/friends. Great people. Very, very supportive. But at the end of the day you also feel a sense of pressures . . . ‘We’ll get you through it’, also comes from, ‘What the hell have you done?’ ‘You’ll definitely get past this’, also comes with, ‘What the hell are you going to do after this?’ Suddenly you have to make promises that you’ll be careful the next time. You’re wondering why? It’s a screw up sure, it’s a hindrance. Suddenly I don’t want to be accountable to everybody about how I’m going to watch my sex life. Be careful. Sure, I know, okay fine I should. But suddenly I felt also like everybody was like, ‘Oh, now are you using condoms?’ Suddenly it’s like I was under scrutiny every time, or I would be.”*—*Aquafina (Chennai, 27 years, non-conforming, pansexual)
	(c) “And again, women will stand by you. This friend of mine, and at this point I was living at home with my parents . . . And I hadn’t the access to the kind of money I needed, so this friend loaned me the entire amount, went with me, sat by me, was amazing.”*—*Jia (Kolkata, 42 years, nonbinary, pansexual)
	(d) “So, one particular time, and it wasn’t even like we weren’t using protection but according to him, the condom slipped. So, I didn’t really pay that much attention, which was stupid I guess, because . . . and like I said, there weren’t emergency measures available. And then I found out I was pregnant. I called him and he’s like, ‘So, what are you going to do about it?’ Like, ‘Okay, so what are we going to do?’ ‘What are you going to do?’ . . . fuck off. I will do what I need to do.”*—*Shivvie (Chennai, genderfluid)
	(e) “I’ve had an abortion before, so my ex-boyfriend actually asked me whether I wanted to keep the child, which is a great thing. I think it was very kind of him to ask at least. He wasn’t like ‘Abort it right now’ or something like that, but he asked me.”*—*Pooja (Bangalore, 22 years, genderfluid, homosexual)
3. Anticipated Judgment	(a) “I was with my ex-boyfriend . . . Usually they would pull it [their penis] out before. But we always had that scare, ‘What if anything went in?’ Like, ‘Oh my god!’ So that day, I went and I was trying to get an I-pill [emergency contraception]- the pill that you take within 24 hours. I tried to get that, and nobody knew what that was. And I’m like, ‘What? It comes on TV all the time! Why do you not have it?’ And they’re like, ‘No, we don’t have it.’ I went to a lot of pharmacies, which was really weird. And I was pretty young back then, and so everybody was like, ‘Hm.’ It was really weird to get them. So I was like, ‘Okay, I’m not able to get the pill, and even if I had to get the pill, it was really expensive, couldn’t afford it.’ So I was like, “Okay, fuck it. Let me just take a pregnancy test.” So, the pee stick. So I just got that, and . . . I went way far away from my house . . . to get one . . . and then I went to my friend’s house and I tried and I’m like, ‘Okay. I’m not pregnant. I don’t trust it.’ It was my first ever pregnancy test, I really don’t know how that works.”*—*Annette (Chennai, 28 years, cisgender female, pansexual)
	(b) “To make things worse, it is a walk of shame to the OB-GYN if you are young, and unmarried. My personal experience, where I was very worried that I might be pregnant, due to my anxiety triggers. The receptionist refused to let me fill out my form, asked for my husband’s name, and I said that I wasn’t married. They asked for my father’s name, and contact number after that, which I felt was very hurtful, and I told them that I was not going to give them that, because it isn’t mandated that I need a guardian’s approval. They could have my name, contact details, and age, and an ID card for verification of my legal age. I was put through what felt like ostracization very clearly, and they were talking amongst themselves, remarking that I might be pregnant before marriage.”*—*Chennai Focus Group Participant (24 years, largely a woman, non-heterosexual)
	(c) “Jia: Well, we had to go to like three different doctors because the first two, well, the one thing that gynecologists still do, is they’ll ask you if you’re married. Because that automatically is the question that means, ‘Are you sexually active?’ If you’re not married, they will not tell you anything about sexual health at all. So the first two, when they heard that I wasn’t married, wanted to have the father there. Interviewer: Was that for permission? Jia: Yeah. So the third person luckily was at one of these hospitals and either she was really busy or didn’t care or something, but that worked out. So she was like, ‘Yeah, okay. We’ll schedule this when you want to do it.’ [In India] everybody’s business is everybody’s business. So everybody from the nurses to the people who were in charge of prepping me for the surgery, the people in charge of shaving stuff and all of that, everybody wanted to know what was going on, why are you here, and what is the procedure? ‘Oh, that is a baby. The father doesn’t want it?’ . . . Now, pretty much everyone knows . . . But for a long time, I just, it’s not something ‘good women’ talk about.”*—*Jia (Kolkata, 42 years, nonbinary, pansexual)
	(d) “I realized it was like a whole bunch of procedures [for an abortion], and all the things that happens to you in a gynaec clinic where the family’s saying your partner has to go with you, and your partner’s more than willing to go with you. But you’re also sitting next to him there and Chennai being Chennai is like—A clinic means at least 10 people you know walk in. You’ve been to school with their children. Somebody you know, some neighbor, some long-lost cousin. Everybody. Then you’re afraid of the clinic, already stressing about yourself, but you’re also stressing about who’s going to find you there with your partner. Whether they think it’s your partner or not, you’re sitting there with a guy. It’s worse enough because what is an unmarried woman doing at a gynaec’s clinic right? That’s the question. Everybody wants to know what you’re doing there. So it’s like, if you’re not pregnant why are you here? That’s when I realized that—we should all be going to the gynaec’s clinic. We should all be getting checked ups.”*—*Aquafina (Chennai, 27 years, non-conforming, pansexual)
	(e) “[When I got the results of the pregnancy test] I was happy. Because I was not ready for a child. As much as I want a child, I was in college and he was being mistreated by his parents, and I was not ready. . .. I did not know how to accept that. At that time was not so much . . . like, now if I get pregnant, I’d be like, ‘Okay, I’m pregnant. Deal with it.’ But at that time, I’m like, ‘Oh, my father’s gonna kill me!’ I think now I’m more confident because my dad’s dead.”*—*Annette’s (Chennai, 28 years, cisgender female, pansexual)
	(f) “What ended up happening is he [my boyfriend] took me to a maternity clinic which is a little far from my house, ’cause we had to get as far away as possible from my place. We can’t be anywhere there, so we went all the way over there and this is the time I was still living with my grandparents . . . After class one day we just went to the maternity clinic and the doctor checked and I was in my first trimester and she was like ‘Two more weeks and I cannot abort this child’ . . . She was like ‘Okay, there are two options. We can either surgically remove it, or we can use the pill, but I will ask you to surgically remove it because it’s a lot less pain and you are a smaller-bodied person and it will get over faster’ and my boyfriend was kind enough to be like ‘I’ll obviously pay for most of it’.”*—*Pooja’s (Bangalore, 22 years, genderfluid, homosexual)

Participants who had experienced a confirmed (such as through a pregnancy test) or unconfirmed unintended pregnancy expressed a number of negative emotional reactions to the situation. Fear, worry and concern, and self-blame were common. This was exacerbated for Aquafina who experienced a second unintended pregnancy (Table 3, 1.1d).

Responding to the fear experienced surrounding an unintended pregnancy, Pompi expressed the desire to take action to avoid another such situation (Table 3, 1.1e).

Similarly, after experiencing an unintended pregnancy in graduate school, Jia became insistent about male partners wearing a condom during sex (Table 3, 1.1f). Jia is describing the care in which they took to taking contraceptive pills but still having a pregnancy and resulting abortion. Karla described less fear due to knowing their menstrual cycle and having a partner helping to prevent pregnancy (Table 3, 1.1 g). Karla also presents a counter viewpoint from others, as abortion is viewed as part of a holistic plan to avoid childbearing.

#### Pregnancy options

Participants generally described not knowing what options were available when first finding out or suspecting they were pregnant, from how to confirm a pregnancy to ways to end the pregnancy. Aquafina was unsure what to do with the pregnancy test (Table 3, 1.2a). Shivvie describes the many processes and decisions that they began when they were pregnant (Table 3, 1.2b). When Karla suspected they might be pregnant, they turned to traditional means of inducing menstruation, rather than seeking medical care through a physician (Table 3, 1.2c).

### Social support (or lack thereof)

In general, many participants operated in isolation while making decisions in relation to an unintended pregnancy without partner or family support. Shivvie described the fear that isolation instilled (Table 3, 2a). Like Shivvie, Aquafina did not know who would be the best person to lend support and described that support was conditional (Table 3, 2b). Beyond conditional support, Aquafina points to others’ involvement in Aquafina’s contraceptive decisions and resulting discomfort. Support was not only emotional but tangible, such as the need for money for abortions (Table 3, 2c).

In the initial stages of decision making, partners were not always supportive as Jia previously described their partner laughing upon finding out about the pregnancy. Shivvie described having “*no idea*” during their first sexual experience when their partner gave them “*the pill that should be taken within 72 hours*” (emergency contraception) and they later missed their period (Table 3, 2d). Pooja’s gratitude when a partner asked them about their own desires in relation to an unintended pregnancy provides evidence for the general lack of support from partners (Table 3, 2e).

### Anticipated judgment

Participants discussed concerns with stigma and surveillance at pharmacies, clinics, as well as from family members. Annette not only faced the barrier of judgment at the pharmacy, but also described a lack of trust in methods of testing for pregnancy (Table 3, 3a). Many participants had minimal to no support through the process and were faced with questions about their husbands and/or fathers when they accessed care for unintended pregnancies (Table 3, 3b).

Jia navigated providers’ stigma about their unmarried status by going to three different providers (Table 3, 3c). Jia described surveillance generally by the community as well as by staff and providers. This surveillance was exacerbated by Jia’s lack of queer community, with few “*female bodied people*” at local queer events and being older than many people, such that Jia didn’t know “*which group to be with*.” Aquafina was also worried about surveillance and acknowledged that cultural silence surrounding sexual health among young and unmarried people worsened the issue (Table 3, 3d). Annette’s father’s anger was one of the most pressing factors in relation to worrying about an unintended pregnancy (Table 3, 3e). Pooja’s concerns about their family’s reactions caused Pooja to seek care far from their home. Pooja’s partner later asked to be repaid for the procedure (Table 3, 3f).

## Discussion

This study explores the understudied area of unintended pregnancy and abortion experiences among SGMF in urban India. Our findings indicate that these experiences are often met with fear and shame, which are compounded by isolation created by lack of support from partners and family, and bias among health care providers. There are similarities with other unmarried women in in India, but the additional stigma from social networks^30,35^ and providers may exacerbate the effects of unintended pregnancy and abortion.

The circumstances surrounding pregnancies within our sample mainly centered around contraceptive issues, including method failure, misuse/non-use, and partner coercion or dishonesty (i.e. participants believed they were using contraception but found out later they were not). SGMF may not see themselves as contraceptive users and use contraception less frequently.^36^ Research has shown that a number of barriers to contraceptive use occur among unmarried people and SGMF in India, including costs, lack of access or knowledge, stigma surrounding sex outside marriage, and medical provider bias.^37,38^ We did not ask about sex of sexual partners, though research has found that SGMF not planning to have penile-vaginal intercourse with males are less likely to use contraception.^39^ Previous work in less urban parts of India reported that one in six unintended pregnancies resulted from nonconsensual intercourse.^11^ Kalyanwala et al.^11^ and Sowmini^16^ describe nonconsensual experiences such as forced or coerced sex as being more prevalent among unmarried young people seeking abortion. Although contraceptive sabotage has been documented to increase the likelihood of abortion among married women in India,^40^ research has yet to address the issue among unmarried young people or SGMF.

The lack of perceived options in the face of an unintended pregnancy was a source of stress for individuals. The Pregnancy Decision-Making Model includes evaluation of values, narratives, and capital, which are influenced by barriers to access and social influence.^41^ Participants in this study discussed their barriers to access and social influences more than their personal evaluations, which may be an artifact of the retrospective nature of the study. Previous research has identified a general lack of awareness among women in India about how to access safe abortions.^42,43^ The use of both allopathic medicine as well as ayurvedic medicine (such as eating specific foods) was reported by participants, though India primarily relies on allopathic medicine.^44^ India’s restrictions on providers’ training and access to facilities for abortion care mean that many ayurvedic providers are not able to provide abortions.^44^ Self-managed abortion was seen as an effective alternative to clinic-based care. In one study of Indian women presenting for a medical abortion, nearly one-third had tried to abort on their own in the last week.^44^ In the current study, ayurvedic methods were the only evidence we found of participants attempting abortion outside of an allopathic system.

Karla’s use of the phrase “almost pregnancy,” when describing using ayurvedic methods to manage a suspected, yet unconfirmed pregnancy, illustrates the overlap between abortion and menstrual regulation. Studies out of Bangladesh and other countries document the use of surgical methods or medication to remove the uterine lining in patients with or without pregnancy confirmation to bring back one’s period.^14,45,46^ Describing suspected pregnancies as “almost pregnancies” may reflect participants’ interest in preventing implantation of the fertilized egg. It may also serve to mitigate stigma associated with unintended pregnancy and abortion, just as the phrase “menstrual regulation” creates the opportunity to reframe abortion in a positive light by addressing how it allows a person to return to typical menstrual functioning.^47^ Alternately, it may “communicate the impermanence of pregnancy without assigning agency to anyone.”^47^

For unmarried women, stigma related to marital status in unintended pregnancy has been documented as a barrier to seeking abortion care.^48,16^ Sexual minority females in India often experience general surveillance related to their sexual identity and behaviors.^29,30^ Additional pregnancy-related surveillance and judgment from providers, family members, as well as others at clinics may be may compound negative assessments of stigma. Previous research with SGMF in the United States describes the challenges of navigating contraception in addition to queer identities, including judgment for having sex with men.^36^ Providers’ judgment or stigma related to marital status reported by participants in this study may be related to providers’ lack of knowledge related to abortion laws and/or fears of liability.^6^ In line with the buffering effects of community belonging, participants in our study mentioned using informal networks of friends and acquaintances to identify potentially supportive medical providers that would act with discretion, suggesting that social networks may play a role in health care seeking behaviors of SGMF.

Disclosure of unintended pregnancy was common among our participants, which has also been found in samples of unmarried Indian women.^11^ As with other studies, participants noted selectively disclosing their pregnancies to those from whom they were most likely to receive support.^11,49^ Still, support from partners involved in the pregnancy was mixed among our sample. Jejeebhoy et al.^9^ and Kalyanwala et al.^11^ found young unmarried women received greater amounts of emotional support (80%) than financial support (55%) from sexual partners. Unmarried people in India may prioritize their career advancement and share abortion decision making between partners.^50^ The presence or lack of social support influenced their reactions and coping with unintended pregnancy and abortion. Participants who felt supported by partners, friends, and family described having help with the logistics surround abortion appointments, as well as help dealing with fear and guilt associated with the pregnancy. Communities of SGM individuals in India can help buffer stigma,^52,51^ though intersectionality can temper feelings of belonging (as Jia points to being older and not feeling included).^53^ Generally, social support mediates the relationship between stressors and health.^54^ In the context of abortion, social support is associated with higher self-efficacy in coping, lower anxiety after abortion, and fewer delays in accessing abortion.^11,53–57^ None of the participants discussed their gender or sexual identity specifically in relation to unintended pregnancy; potentially they were presenting more cisgender and/or in monogamous partnerships with cisgender men.

### Strengths and limitations

This study expands upon the literature about unintended pregnancy and abortion among unmarried people in India and provides a glimpse into those experiences among SGMF. Although the research team was primarily from the United States, our partnership with community organizations facilitated recruitment and contextualization of findings. Individuals in this study were connected to sexual and gender minority-serving organizations, which may also indicate higher levels of access to resources in general. Data saturation related to unintended pregnancy was not reached in this sample, as the study was not primarily focused on unintended pregnancy and abortion experiences. It is possible that additional themes specific to identifying as a sexual- or gender-minoritized person were not captured here. Therefore, future research should explore this topic among SGMF more in-depth.

## Conclusion

These findings highlight the effects of stigma in relation to abortion and unintended pregnancy on health and relationships. Many of participants’ experiences are similar to studies on heterosexual and cisgender women, though we find evidence of stigma operating in unique ways for SGMF.

## Supplementary Material

Supplementary material
