# Supplementary material for: “It’s a walk of shame”: Experiences of unintended pregnancy and abortion among sexual- and gender-minoritized females in urban India
Source: Med Access Point Care. 2021 Jul 31;5:23992026211027698. doi: 10.1177/23992026211027698 (PMC9413609; doi:10.1177/23992026211027698)
Supplement: Supplementary material [file sj-docx-1-map-10.1177_23992026211027698.docx]

## Data Extraction Format

1. PATIENT DEMOGRAPHIC INFORMATION
2. Age_____ gender________ year and season of admission______
3. Migration status: migrant_________ resident___________
4. Migration origin in administrative zone_____________
5. Migration site_______________
6. Clinical information
7. Duration of illness___________
8. Presence of clinical symptoms

Fever _______ weightloss _______ voimiting/diarrhea______ bleeding________

1. Presence of clinical signs

Jaundice lymphadenopathy spleenomegally and size______ ascites

1. Laboratory profiles

Wbc________

Hgb_________

Plt___________

HIV status: non-reactive reactive unknown

1. Presence of coinfection: yes no

If yes; tuberculosis malaria pneumonia neutropenic sepsis

1. Leishmania diagnosis
2. Leishmania status: new relapse
3. Rk39 : positive negative unkown
4. Tissue aspiration

Positive negative inconclusive not done
